# The “Fortilat” Randomized Clinical Trial Follow-Up: Neurodevelopmental Outcome at 18 Months of Age

**DOI:** 10.3390/nu12123807

**Published:** 2020-12-11

**Authors:** Chiara Peila, Elena Spada, Sonia Deantoni, Ester Iuliano, Guido E. Moro, Marzia Giribaldi, Laura Cavallarin, Francesco Cresi, Alessandra Coscia

**Affiliations:** 1 Department of Public Health and Pediatric, Neonatal Unit of Turin University, Via Ventimiglia 3, 10126 Turin, Italy; elenaspada.bios@gmail.com (E.S.); deantoni.so09@gmail.com (S.D.); ester.iuliano@unito.it (E.I.); francesco.cresi@unito.it (F.C.); alessandra.coscia@unito.it (A.C.); 2 Italian Association of Human Milk Banks, Via Libero Temolo 4, 20126 Milan, Italy; guidoemoro@tiscali.it; 3 Institute of Sciences of Food Production, National Research Council, Largo Braccini 2, 10095 Grugliasco, Italy; marzia.giribaldi@ispa.cnr.it (M.G.); laura.cavallarin@ispa.cnr.it (L.C.); 4 Research Centre for Engineering and Agro-Food Processing, Council for Agricultural Research and Economics, Strada delle Cacce 73, 10135 Turin, Italy

**Keywords:** human milk, human milk fortifier, donkey milk, adjustable fortification, VLBW infants, preterm infants, neurodevelopment outcome, GQ

## Abstract

Adequate nutrition is fundamental to neonatal survival and short-term outcomes, but it also has long-term consequences on quality of life and neurologic development of preterm infants. Donkey milk has been suggested as a valid alternative for children allergic to cows’ milk proteins, due to its biochemical similarity to human milk; we, hence, hypothesized that donkey milk could be a suitable basis for developing an innovative human milk fortifier for feeding preterm infants. The aim of the current study was to extend the findings and to evaluate the neurodevelopmental outcomes at 18 months of corrected age of the infants enrolled in the clinical trial named “Fortilat”. Infants born ≤1500 g and <32 weeks of gestational age were randomized to receive either a combination of bovine milk-based multicomponent fortifier and protein supplement or a combination of a novel multicomponent fortifier and protein supplement derived from donkey milk. The followed fortification protocol was the same for the two groups and the two diets were designed to be isoproteic and isocaloric. All infants enrolled were included in a developmental assessment program. The neurodevelopmental assessment was performed at 18 ± 6 months of corrected age. Minor and major neurodevelopmental impairment and General Quotient (GQ) at the Griffiths-II Mental Development Scale were considered. The GQ was considered both in continuous and as two classes: lower than and higher than (or equal to) a defined cutoff (GQcl). The difference in GQ and GQcl between the two arms was estimated using Mann–Whitney–Wilcoxon test or Fischer exact test, respectively, on the assumption of casual loss at follow-up. A further analysis was performed using generalized linear models. There were 103 children (bovine milk-derived fortifier arm = 54, donkey milk-derived fortifier arm = 49) included for the neurodevelopmental follow-up. All observations were included in the interval of 18 ± 6 months of corrected age. No significant difference was observed between the two arms in the incidence of neurologic sequelae and the GQs were similar in the two arms. Our results demonstrated no difference for the donkey milk-derived fortifier compared to standard bovine-derived fortifier regarding long-term neurodevelopmental outcomes.

## 1. Introduction

Very preterm newborns (gestational age (GA) < 32 weeks) and very low birth weight infants ((VLBW) birthweight ≤ 1500 g) are at risk of inadequate growth and short- and long-term sequelae, in part due to prematurity and in part due to comorbidities [1]. Neurodevelopmental impairment, a significant long-term complication associated with preterm birth, is generally defined as the presence of one or more of these features: cognitive delay, cerebral palsy, or hearing or visual impairment. In addition, also behavioral, psychological, and functional outcomes can greatly impact the quality of life of these patients and have been increasingly considered as significant in outcome studies. Multiple factors can influence neurodevelopmental outcomes, such as GA at birth, size for GA, brain injury, growth, neonatal morbidities, and parental socioeconomic status [2]. Nutrition represents a chance to promote an adequate growth and neurologic development [3,4,5]. Well established is the link between early nutrition and neurodevelopmental impairment, and this can be explained by the sensitivity of the developing brain to nutrition [5]. Increased macronutrient and energy intake in the first weeks after birth seems to be associated with better neurodevelopmental outcomes, such as improved language score in VLBW babies, increased developmental quotient in extremely preterm babies, and lower incidence of brain lesions in babies <30 weeks of GA [6]. Nonetheless, early recommended nutrient intakes are frequently not achieved and postnatal undernutrition and growth failure are still common. On this focus, the European Milk Bank Association (EMBA) highlights that achieving an optimal growth and adequate nutrition are the main targets for a successful management of preterm infant care [7,8]. The main issue in clinical practice is to ensure an adequate qualitative and quantitative nutrition, particularly in terms of protein intake [7]. Although human milk (HM) is undoubtedly the gold standard of nutrition for every newborn, in the case of premature birth it is inadequate for the nutritional needs of infants since it provides insufficient amounts of several nutrients [9,10,11]. HM must, therefore, be fortified with the nutrients in short supply [12,13]. During the last decade new fortification strategies and different commercially available fortifiers have been developed and studied. Nevertheless, the optimal method for HM fortification remains to be determined and a variety of protocols are currently used [14,15,16,17,18]. Recently, human milk-based fortifiers have been proposed, but their utilization is limited by high costs and ethical issues. Moreover, there is no strong evidence that human milk-based fortifiers in otherwise exclusively human milk-fed preterm infants affect important outcomes [13]. In this context, Coscia et al. hypothesized that donkey milk could be a suitable basis for developing an innovative human milk fortifier and conducted a randomized clinical trial named “Fortilat” [19,20].

Milk from monogastric animals, rather than from ruminants, has been suggested during recent years to be more suitable for human nutrition based on its physiochemical properties, including more similar protein and lipid compositions to that of human milk [21,22]. Donkey milk showed biological effects comparable with those elicited by human milk and it has a protein profile more similar to that of human milk in terms of relative abundance and primary structure in comparison with bovine milk [19,23]. In addition, it has been demonstrated in murine models that a supplementation of the basal diet with donkey milk decreases the accumulation of body lipids and affects glucose and lipid metabolism in a manner more similar to human milk than cow milk [24].

We hypothesized that such differences may impact the protein utilization in preterm infants and, consequently, that donkey milk may be more suitable than bovine milk as an ingredient in human milk fortifiers. Our study evaluated the feeding tolerance, growth, and clinical short-term outcomes in a population of preterm infants fed with a novel multicomponent fortifier and a protein concentrate derived from donkey milk (DF), in comparison to an analogous population fed with a traditional fortifier and a protein supplement containing bovine milk proteins (BF) [19,20]. All infants received isocaloric and isoproteic supplementations of HM (according to the adjustable fortification protocol) (ADJ).

The aim of the current study was to extend these findings and evaluate the neurodevelopmental outcomes at 18 months of age of the “Fortilat” trial.

## 2. Materials and Methods

### 2.1. Clinical Trial and Intervention

The study was performed in the Neonatal Intensive Care Unit of the University, City of Health and Science of Turin; it was registered (http://www.isrctn.com/ISRCTN70022881, ISRCTN70022881) and approved by Local Ethic Committee (AN: 0025847, 27/05/2014).

Recruitment period was between 27 November 2014 and 22 December 2016.

The inclusion criteria were: GA <32 weeks or birthweight ≤1500 g, exclusive feeding with human milk (fresh own mother’s or donor milk), and enteral feeding ≥80 mL/kg/day of human milk reached within the first four weeks of life. Neonates affected by severe gastrointestinal pathologies (such as necrotizing enterocolitis, colostomy, intestinal obstruction, symptoms of peritonitis, presence of blood in the feces), chromosomal abnormalities or major malformations, hereditary metabolic diseases, intravascular disseminated coagulopathy (IDC), shock, patent ductus arteriosus (PDA) requiring medical care or surgery at time of randomization, and severe renal failure (serum creatinine >2 mg/dL) were excluded. After informed, written parental consent was obtained, infants were randomized 1:1 by a software-generated list in one of the following groups. The control group (BF-arm) underwent fortification with a multicomponent fortifier and a protein concentrate derived from bovine milk. The Fortilat-group (DF-arm) underwent fortification with a multicomponent fortifier and a protein concentrate derived from donkey milk. Please refer to our previous papers for a detailed description of the study protocol [19,20]. Briefly, the experimental products were produced by ultrafiltration of pasteurized donkey milk in a pilot, stainless-steel plant. Retentates from the ultrafiltration processes were then pasteurized and aseptically lyophilized and packed. All the batches used for the trial were analyzed for the microbiological and chemical profile and complied with the safety criteria required by Italian legislation. The products were stored at −80 °C until used. All newborns received enteral feeding according to a regimen of adjustable fortification, based on blood urea nitrogen determination, for 21 days. The intervention started when the infants were able to tolerate a volume of ≥80 mL/kg/day (randomization time) and, according to study protocol, was planned to last 21 days; the intervention was suspended at discharge from the hospital for any reason (transfer, death, discharge home). Babies were discharged from the hospital when they met all the following criteria: satisfactory weight gain while receiving full oral feeding, maintenance of adequate thermal stability, and resolution of acute medical conditions.

### 2.2. The Fortilat Follow-Up Protocol

All infants enrolled in the trial were included in a developmental assessment program that consisted of hospital visits at 40 ± 1 week of postmenstrual age and at 6, 12, and 18 months of corrected age. At each visit, medical history was taken and growth evaluation was performed. Physical and neurological examinations were performed by an experienced neonatologist in the follow-up program.

Regarding auxological parameters, weight, length, and head circumference measurements were taken and recorded according to standard anthropometric procedures. The neonatologists took measurements using identical equipment: an electronic scale (Seca, Hangzhou, China) for weight, a specially designed Harpenden infantometer (Chasmors, London, UK) for length, and a metallic non-extendable tape (Chasmors) for head circumference. The equipment, which was calibrated twice a month, was selected for accuracy, precision, and robustness. Measurement procedures were standardized on the basis of WHO recommendations to ensure maximum validity [25,26].

The neurodevelopmental assessment was performed by a multidisciplinary team, with a standardized protocol, at 18 ± 6 months of corrected age; minor neurodevelopmental impairment, major neurodevelopmental impairment, and General Quotient (GQ) at the Griffiths-II Mental Development Scale (GMDS) were considered. The Griffiths-II Mental Developmental Scale assesses different developmental areas (fine motor, gross motor, language, cognitive, and personal-social-emotional) using five scales (A–E) for children between 0 and 2 years of age: the Locomotor Scale, the Personal-Social Scale, the Hearing and Speech Scale, the Eye and Hand Coordination Scale, and the Performance Scale. The test provides a subquotient for each scale and an overall General Quotient (GQ). Presence of minor impairment was evaluated by a trained developing age specialist and was intended as the presence of at least one of the following: mild motor impairment condition that limits the child in learning and adaptation, i.e., slight reduction in intellectual performance; perceptual-motor development defects resulting in difficulties in balance and coordination; a motor hindrance; gross or fine motor coordination difficulties; muscle tone imbalance, but without definite signs of cerebral palsy; lower verbal expression skills than expected; or minor visual defect impairment (i.e., strabismus, nearsightedness, or refractive defects diagnosed by a pediatric ophthalmologist) [27]. Major neurodevelopmental impairment was defined as the presence of at least one of the following: cerebral palsy (according to the Executive Committee for the Definition of Cerebral Palsy definition), blindness (i.e., total or severe unilateral or bilateral visual impairment), deafness (i.e., need for unilateral or bilateral hearing systems), or a GQ < 70 [28].

### 2.3. Subjects and Statistical Analysis

The randomized, controlled, clinical trial included 156 subjects: BF arm *n* = 79, DF arm *n* = 77. The present research included only the children with available follow-up visit at 18 months of Corrected Age (CA). Weight, head circumference, and length at birth were expressed in z-score using neonatal Intergrowth21st [29] and INeS charts [30]. The z-score values lower than –4 or higher than +4 were considered outliers. The children having birth weight lower than the 10th or higher than the 90th centile were defined Small for GA (SGA) or Large for GA (LGA), respectively. The GQ, expressed in % and defined as 100 × development quotient (months)/corrected age (months), was considered in continuous and as two classes. Since no child resulted in a GQ lower than 70 (the cutoff defined as reference), the first quartile (q1) of GQ distribution in the control (BF) arm was used as a cutoff to create the two classes: GQcl. The difference in GQ and GQcl between the two arms was estimated using Mann–Whitney-Wilcoxon test or Fischer exact test, respectively, on the assumption of casual loss at follow-up. A further analysis was performed using linear regression for GQ and logistic regression for GQcl. Arm, birth head circumference z-score (according to neonatal Intergrowth21st or INeS charts), bronchopulmonary dysplasia (BPD), minor neurological sequelae, GA at birth, and segmented on population (GA at birth <32 weeks, GA at birth ≥32 weeks with birth weight <1501 g) were included in the models as covariates. The last covariate was included because the double inclusion criteria (all neonates with GA <32 weeks and neonates with GA ≥32 weeks only if birth weight is less than 1501 g) defined two different groups (or populations) of neonates. The first population (GA <32 weeks) included neonates with a higher risk of morbidities related to low GA, whereas the second population included a higher proportion of females and twins (both physiologically smaller), intrauterine growth restriction (IUGR) babies (pathologically smaller), and SGA babies (smaller by definition). To normalize the GQ distribution, the best power parameter of Box–Cox transformation was detected and the GQ transformed values were used in the regression model. Data analysis was performed with SAS^®^ software version 9.4 (Copyright (c) 2016 by SAS Institute Inc., Cary, NC, USA).

## 3. Results

Of the 156 children included in the previous study, 53 (BF-arm = 25, DF-arm = 28) were excluded since information at 18 months regarding birth head circumference, major and minor neurological sequelae, or GQ value was not available. At birth, we found one value of length z-score equal to −4.04 according to Intergrowth21st neonatal charts, but it was not considered as an outlier since the z-score according to the INeS charts was equal to −3.31. No other outliers were detected.

Table 1 reports the basal characteristics of the 103 children (BF-arm = 54, DF-arm = 49) included in this study. No neonates presented necrotizing enterocolitis (NEC) and periventricular leukomalacia (PVL). A total of nine newborns were affected by intraventricular hemorrhage. The percentage of respiratory distress syndrome, BPD, intraventricular hemorrhage, retinopathy of prematurity, and patent ductus arteriosus was higher in the BF-arm where the percentage of neonates with GA <32 weeks was higher, while in the DF-arm a higher percentage of fetal growth restriction was observed. One child had major neurological sequelae and 16 children had minor neurological sequelae. A total of 12 infants had BPD. Regarding auxological variables at birth, the mean z-score was higher in the BF-arm than in the DF-arm. This is due to the higher percentage of children with GA <30 weeks included in the BF-arm than in the DF-arm. The median (interquartile range (IQR)) corrected age at the neurological follow-up was 18 [17,18,19] months in BF-arm and 18 [17,18,19,20] months in DF-arm, while the median postnatal age was 21 [20,21] and 20 [19,20,21,22] months, respectively. All observations were included in the interval of 18 ± 6 months of correct age.

### 3.1. GQ in Continuous

**Figure 1:**
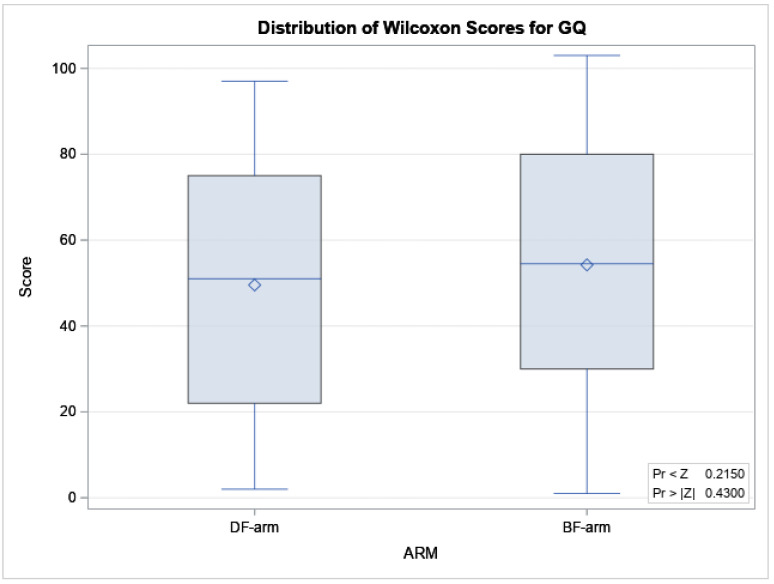
Box plot of Wilcoxon score by arm.

The median (interquartile range) GQ was 101.6 (97.1–104.4) in the BF-arm and 101.2 (93.6–103.8) in the DF arm. Figure 1 shows the box plot of Wilcoxon scores classified by arm. No difference between the two arms was detected (*p* = 0.430).

The Box–Cox transformation having λ = 1.35 resulted in the best to normalize GQ distribution. The difference between the two arms was not significant either when head circumference z-scores were computed according to neonatal Intergrowth21st charts or when they were computed according to INeS charts: the least square means (after retro-transformation) differed by about 2.4 points (*p* = 0.19). Table 2 reports the estimates of linear regression coefficients. As expected, minor neurological sequelae significantly reduce the GQ at 2 years.

### 3.2. GQ in Two Classes

The cutoff to define the two classes, corresponding to the first quartile (q1) of GQ distribution in the control (BF) arm, was 97.076.

Table 3 reports the frequencies of children by arm and GQcl. No difference in the distribution of values <q1 in the two arms were detected (Fisher’s exact test: *p* = 0.3843) and the odds ratio (confidence interval (95%)) resulted in being 1.529 (0.645; 3.626). Similar results were observed with the logistic regression. Table 4 reports the estimate of Odds Ratio (OR) (confidence interval (95%)) for arm and for the other covariates included in the model: No differences were observed between arms, and minor neurological sequelae resulted in being a risk factor for low GQ score at 18 months of age.

## 4. Discussion

Inadequate nutrition and/or poor postnatal growth have been reported as negatively associated with neurocognitive outcomes in preterm infants [5,6]. However, the data existing in literature are mainly concentrated on the evaluation of the effects of different quantitative protein intake on the long-term outcomes [31,32,33,34]. Our study is one of the few that evaluated the effects of different qualitative protein intakes. In fact, the “Fortilat” study was a randomized, controlled, clinical trial that assessed the effects of a donkey milk-derived human milk fortifier vs. a bovine milk-derived human milk fortifier among very preterm newborns and VLBW infants [19,20]. We speculated that the quality of donkey milk proteins could be responsible of our previous finding regarding the feeding tolerance being better in the DM arm, since the two diets were isoproteic and isocaloric and all newborns, in both arms, received exclusively human milk (raw own mother’s milk or pasteurized donor milk), without any preterm formula.

The current study evaluated, among long-term outcomes, a different and important aspect that may be influenced by the quality of nutrition: the neurodevelopmental outcomes at 18 months of age. No significant differences were observed between the two arms in the incidence of neurological sequelae, and the mean GQ was similar in the two arms. Moreover, the two arms did not differ in the probability of having GQ < cutoff (1st quartile of control arm). These results were confirmed also after correction for birth head circumference z-score (according to neonatal Intergrowth21st or INeS charts), GA at birth, BPD, and minor neurological sequelae. Our data reflected our expectation: In this context, our aim was not to show the superiority of the novel donkey-derived fortifier as much as to show no differences compared to the standard bovine-derived fortifier. In fact, it is important to highlight that all our babies received breast milk, both maternal or donated, as the main source of nutrition, and human milk has been proven to have a positive effect on neurodevelopmental outcomes. It would have been difficult to appreciate differences between the two arms in a population that already had such a positive factor influencing their neurodevelopment.

In addition, our population of very preterm and VLBW infants had, as expected for such a population, many risk factors for neurodevelopmental impairment so that the difference between the two fortifiers on later outcomes is difficult to be appreciated as the impact of nutrition is less evident. Dividing the population in risk classes could help highlight eventual positive effects, but our sample was not large enough to further explore such possibility. Moreover, since our initial sample of enrolled patients was reduced during the follow-up for missing information at 18 months of CA, we coincidentally found a higher percentage of babies classified as small for gestational age in the DF-arm comparing to the BF-arm, for weight as well as for head circumference and length. This should reassure about our findings since being SGA is a risk factor for minor neurodevelopmental impairment and, nonetheless, the DM-arm showed similar scores to the BM-arm.

A limitation of our study is that we were unable to collect information regarding body composition. The effects of nutritional interventions on growth are less easily demonstrated from a quantitative point of view (i.e., weight, length, or head circumference growth) than a qualitative point of view (i.e., in term of body composition). Body composition better describes the quality of growth and appears to be linked with neurodevelopmental outcomes. In particular, higher rates of weight and fat-free mass gains between term and 4 months of corrected age are associated with better speed of processing at ca 4 months and 4 years of age [35,36]. Moreover, the study protocol has not been designed to assess outcomes besides the primary endpoints of the study (i.e., feeding intolerance during the observational period). Further analysis could examine if the donkey-derived milk fortifier, with a protein and fat profile more similar to the human milk compared to the bovine milk, may lead to a different pattern of growth in terms of fat mass and fat-free mass accretion, thus influencing the neurodevelopmental outcome.

## 5. Conclusions

This is the first study investigating the use of a donkey milk-based human milk fortifier for feeding very preterm and VLBW infants. Our data showed that donkey milk-derived fortifiers improve the feeding tolerance in preterm infants when compared with standard bovine-derived fortifiers: The DF reduced the occurrence of episodes of feeding intolerance, feeding interruptions, bilious gastric residuals, and vomiting [19]. It is important to highlight that the best tolerance was observed in the DF-arm, in which SGA subjects, at major risk for feeding difficulties, were more numerous. The use of DF also reduced the frequency of gastroesophageal reflux in infants showing clinical signs of gastroesophageal reflux and cardiorespiratory symptoms associated to feeding intolerance [37]. A recent urinary metabolomics investigation revealed that the different quality of the nutrients provided resulted in different urinary metabolic patterns [38]. Moreover, we also reported that DF- and BF-arms had similar short-term growth outcome as well as long-term auxological and neurodevelopmental outcomes.

The results of this study may constitute a basis on which to plan a further multicenter trial to confirm the higher tolerability of the donkey milk-derived fortifiers and their non-inferiority in terms of growth and neurodevelopmental outcomes.

## Figures and Tables

**Table 1:** Basal characteristics of the 122 children included in the study.

		BF-arm *N* = 54	DF-arm *N* = 49
Boys	*n* (%)	25 (46.3)	24 (49.0)
GA < 32 weeks	*n* (%)	44 (81.5)	33 (67.4)
GA (days)	median (IQR)	209 (193–218)	221 (210–226)
Minor Neurological Sequelae	*n* (%)	7 (13.0)	9 (18.4)
Major Neurological Sequelae	*n* (%)	0 (0.0)	1 (2.0)
Broncho Pulmonary Dysplasia	*n* (%)	9 (16.7)	3 (6.1)
RDS	*n* (%)	47 (87.0)	40 (81.6)
Intraventricular hemorrhage	*n* (%)	6 (11.1)	3 (6.1)
Periventricular leukomalacia	*n* (%)	0 (0.0)	0 (0.0)
Patent Ductus Arteriosus	*n* (%)	17 (31.5)	7 (14.3)
Fetal Growth Restriction	*n* (%)	17 (32.1)	25 (51.0)
Retinopathy of prematurity	*n* (%)	9 (16.7)	4 (8.2)
Necrotizing Enterocolitis	*n* (%)	0 (0.0)	0 (0.0)
Length of stay	median (IQR)	44.5 (32–70)	35 (29–51)
Birth head circumference *			
cm	mean (SD)	26.3 (2.29)	27.0 (2.12)
z-score Int21s	mean (SD)	−0.54 (1.00)	−1.07 (1.04)
<10th centile Int21st	*n* (%)	16 (30.2)	21 (42.9)
>90th centile Int21st	*n* (%)	1 (1.9)	0 (0.0)
z−score INeS	mean (SD)	−0.32 (1.07)	−0.97 (1.13)
<10th centile INeS	*n* (%)	12 (22.2)	20 (40.8)
>90th centile INeS	*n* (%)	4 (7.4)	2 (3.9)
Birth length **			
cm		36.6 (3.03)	37.5 (2.73)
z-score Int21s	mean (SD)	−1.00 (0.87)	−1.59 (1.23)
<10th centile Int21st	*n* (%)	15 (35.7)	23 (59.0)
>90th centile Int21st	*n* (%)	0 (0.0)	0 (0.0)
z-score INeS	mean (SD)	−0.58 (1.00)	−1.26 (1.28)
<10th centile INeS	*n* (%)	11 (25.6)	20 (51.3)
>90th centile INeS	*n* (%)	1 (2.3)	2 (5.1)
Birth weight			
g	mean (SD)	1116 (310.0)	1192 (290.5)
z-score Int21s	mean (SD)	−0.78 (1.13)	−1.37 (1.12)
<10th centile Int21st (SGA)	*n* (%)	21 (39.6)	27 (55.1)
>90th centile Int21st (LGA)	*n* (%)	1 (1.9)	0 (0.0)
z-score INeS	mean (SD)	−0.41 (1.10)	−1.03 (1.06)
<10th centile INeS (SGA)	*n* (%)	14 (25.9)	21 (42.9)
>90th centile INeS (LGA)	*n* (%)	3 (5.6)	0 (0.0)

BF-arm: preterm infants randomized on adjustable fortification with bovine milk-based supplements; DF-arm: preterm infants randomized on adjustable fortification with donkey milk-based supplements; IQR: interquartile range; SD: standard deviation; GA: gestational age; SGA: small for gestational age (birthweight < 10th centile); LGA: large for gestational age (birthweight >90th centile); RDS: respiratory distress syndrome; * *n* = 106 (BF-arm = 54, DF-arm = 52); ** *n* = 88 (BF-arm = 44, DF-arm = 44).

**Table 2:** Estimate of linear regression coefficients.

Parameter	Intergrowth 21st		INeS	
	Estimate ± SE	*p*	Estimate ± SE	*p*
Intercept	302.5 ± 94.5	0.0019	291.7 ± 91.9	<0.0001
Arm: DF vs. BF	−11.9 ± 8.9	0.1853	−11.9 ± 8.9	0.1857
Birth head (z−score)	1.77 ± 4.84	0.7160	1.78 ± 4.38	0.6857
BPD: yes vs. no	−4.30 ± 16.19	0.7911	−5.17 ± 15.94	0.7466
Minor neurological sequelae: yes vs. no	−35.0 ± 12.2	0.0051	−34.2 ± 12.0	0.0053
GA (weeks)	2.84 ± 2.90	0.3310	3.15 ± 2.83	0.2680
Population: GA < 32 wks vs. GA ≥ 32 wks	−4.29 ± 14.8	0.7733	−3.22 ± 14.4	0.8236

**Table 3:** Absolute frequencies of General Quotient values lower than and higher than (or equal to) the cutoff (97.076), defined as the first quartile (q1) of the BF-arm distribution.

	<Q1	≥Q1
BF-arm	13	41
DF-arm	16	33

**Table 4:** Odds ratio (confidence interval (95%)) for General Quotient score lower than the cutoff (97.076), defined as the first quartile (q1) of the BF-arm distribution.

	Intergrowth21st		INeS	
	OR [CI (95%)]	*p*	OR [CI (95%)]	*p*
Arm: DF vs. BF	1.887 (0.665; 5.352)	0.2325	1.941 (0.680; 5.540)	0.2152
Birth head (z-score)	0.832 (0.476; 1.452)	0.5171	0.898 (0.545; 1.480)	0.6743
BPD: yes vs. no	2.007 (0.371; 10.863)	0.4190	2.312 (0.437; 12.223)	0.2824
Minor neurological sequelae: yes vs. no	4.319 (1.267; 14.726)	0.0194	3.930 (1.174;13.159)	0.0264
GA (weeks)	0.872 (0.625; 1.215)	0.4173	0.839 (0.609; 1.156)	0.3239
Population: GA < 32 wks vs. GA ≥ 32 wks	0.655 (0.121; 3.546)	0.6232	0.520 (0.102;2.660)	0.4327
